# Case report: ^18^F-FDG PET/CT in pulmonary enteric adenocarcinoma

**DOI:** 10.3389/fonc.2024.1447453

**Published:** 2024-10-14

**Authors:** Zhe-Huang Luo, Xiao-Yan Luo, Xiu-Qin Luo, Ai-Fang Jin, Qing-Yun Zeng

**Affiliations:** ^1^ Department of Nuclear Medicine, Jiangxi Provincial People’s Hospital, the First Affiliated Hospital of Nanchang Medical College, Nanchang, China; ^2^ Clinical Laboratory, Jiangxi Provincial Children’s Hospital, Children's Hospital Affiliated to Nanchang Medical College, Nanchang, China; ^3^ Cardio-Thoracic Surgery, Jiangxi Provincial People’s Hospital, The First Affiliated Hospital of Nanchang Medical College, Nanchang, China

**Keywords:** pulmonary enteric adenocarcinoma, ^18^F-fluordeoxyglucose, positron emission tomography/computed tomography, diagnosis, case report

## Abstract

Pulmonary enteric adenocarcinoma (PEAC), an uncommon variant of lung cancer, presents significant diagnostic challenges due to its overlapping characteristics with colorectal adenocarcinomas. We present a case of a 55-year-old non-smoking female patient diagnosed with PEAC. The patient’s initial symptoms included fever, cough, and sputum production, with air space consolidation on CT, leading to an initial diagnosis of pneumonia. Sputum culture after admission showed no growth of bacteria and fungi. Anti-inflammatory therapy was not ideal. Subsequent bronchoscopy with endobronchial ultrasound and biopsy confirmed the diagnosis of PEAC. Gastroscopy and colonoscopy yielded negative results, and a PET/CT scan revealed an FDG-avid lesion in the right middle lobe, with no other significant hypermetabolic gastrointestinal lesions, thereby excluding an extrapulmonary primary gastrointestinal malignancy. The patient was ultimately staged as PEAC (T4N1M0, stage IIIb). She declined anti-tumor therapy and experienced clinical deterioration during follow-up. This case report expands the radiological spectrum of PEAC, adds to the limited literature, and emphasizes the role of ^18^F-FDG PET/CT in diagnosing such diseases. It also underscores the importance of a multidisciplinary approach in the management of PEAC.

## Introduction

Pulmonary enteric adenocarcinoma (PEAC) represents a rare subtype of non-small cell lung cancer (1), accounting for approximately 0.6% of all primary pulmonary adenocarcinomas (2). It is characterized by adenocarcinomas that are primarily lung-based yet share morphological and immunohistochemical features with colorectal adenocarcinomas, belonging to the histological variants of invasive pulmonary adenocarcinoma. PEAC exhibits several morphological and immunohistochemical features common to both lung cancer and colorectal adenocarcinoma, posing significant challenges in differential diagnosis for clinical pathologists (3). In PEAC, the enteric differentiation component exceeds 50% (4). To confirm the diagnosis of PEAC, colonoscopy and esophagogastroduodenoscopy should be performed to exclude primary gastrointestinal malignancies (5). The diagnosis and differential diagnosis require a careful integration of clinical and pathological data, including endoscopic findings, morphological characteristics of the lesion, and immunohistochemical expression profiles. Additionally, serum carbohydrate antigen 199 (CA199) levels may elevate in PEAC patients.

The ^18^F-FDG PET/CT imaging modality is a whole-body examination and provides a comprehensive assessment of both anatomical structures and metabolic activities. It is highly sensitive in identifying tumors and provides enhanced precision for oncological staging (6). To date, very few PET/CT studies of PEAC have been conducted. Here, we present a case of PEAC from China and review the relevant literature, attempting to enhance the understanding of this disorder and highlighting the role of ^18^F-FDG PET/CT in the diagnostic workup of PEAC, particularly in excluding pulmonary metastatic colorectal adenocarcinoma.

## Case description

On March 29, 2024, a 55-year-old non-smoking female patient was admitted to our hospital with a 1-month history of fever, cough, and sputum production. She denied abdominal pain, vomiting, constipation, change in stool color, or any change in bowel habits. Her initial presentation occurred in late February 2024. At a local hospital; she complained of fever, cough, and expectoration, devoid of chills or hemoptysis. A chest CT scan was performed, which revealed bilateral pneumonia. She was commenced on a 1-week course of piperacillin and tazobactam, resulting in initial symptomatic improvement. A follow-up CT scan on March 5 showed resolution of the inflammation in the left lung; however, the lesion in the right lung had notably progressed. Thereafter, she was administered moxifloxacin for an additional week. Despite this, she experienced a recurrence of symptoms, including fever, cough, and expectoration, which were more intense than her initial presentation on March 26. In light of the exacerbation, she was referred to our hospital for further investigation and management. The patient’s past medical and family histories were unremarkable. The physical examination revealed no significant abnormalities, except for the presence of dry and moist rales upon lung auscultation.

**Figure 1:**
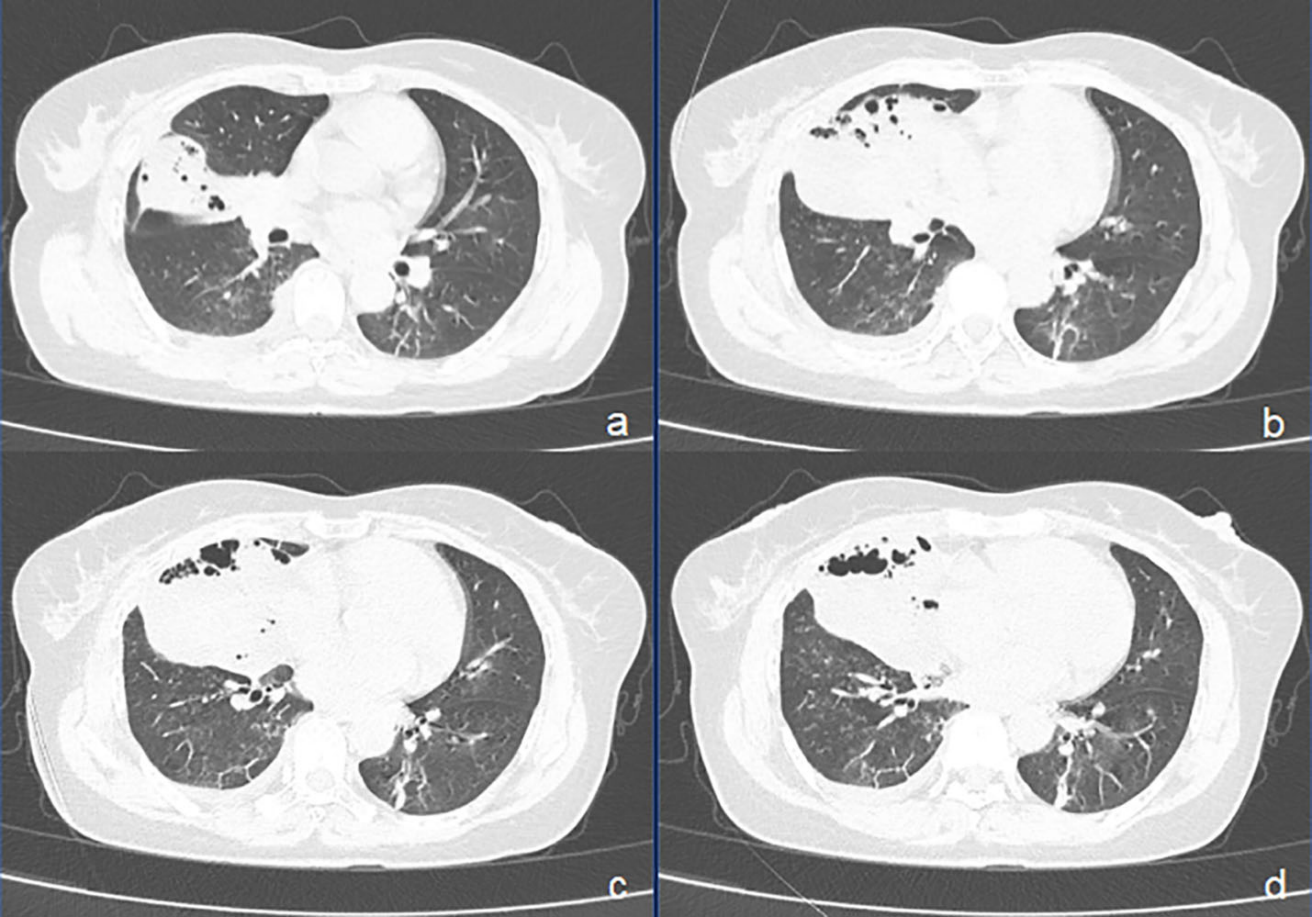
CT images of pulmonary enteric adenocarcinoma (PEAC) showing right middle lobe atelectasis with honeycomb and a small amount of fluid in the right pleural cavity. **(A–D)** axial CT (lung window).

**Figure 2:**
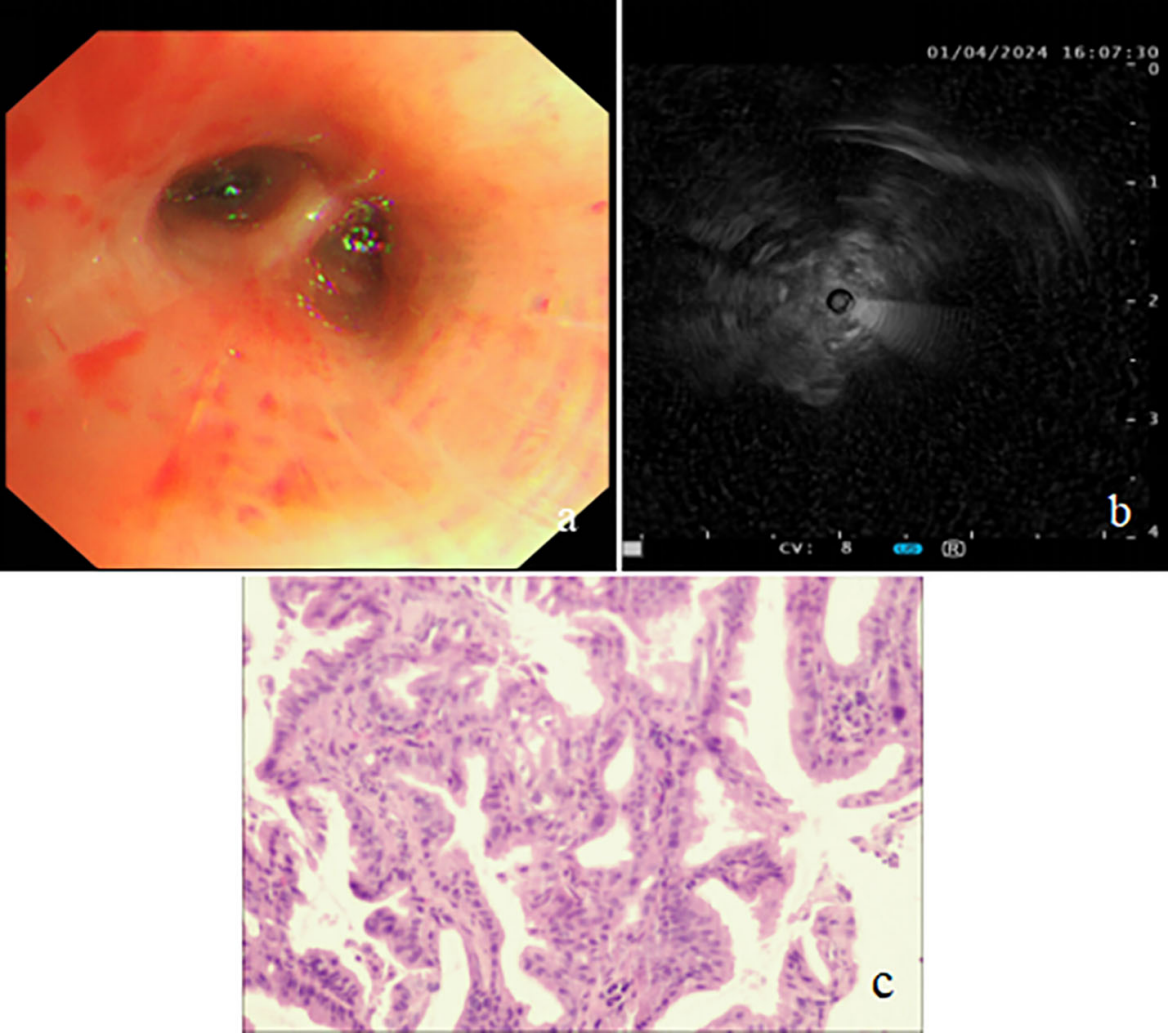
Bronchoscopy findings and biopsy pathology image. **(A)** Bronchoscopy revealing extrinsic compression of the right middle lobe bronchus. **(B)** Endobronchial ultrasound (EBUS) showing a hypoechoic mass in the lateral segment of the right middle lobe. **(C)** Biopsies showing the tumor with glandular architecture (H&E stain, power of magnification ×200).

On admission, the patient’s vital signs were as follows: body temperature of 38.7°C, pulse rate of 91 beats per minute, respiratory rate of 20 breaths per minute, and blood pressure of 109/59 mmHg. Her weight was 57 kg. Laboratory investigations were conducted, and the findings are detailed in **Table 1**. Sputum culture after admission showed no growth of bacteria and fungi. A negative result was obtained for the TB interferon-gamma release assay (IGRA), and her liver and renal function tests were within normal ranges. A chest CT scan performed on March 31 revealed atelectasis of the right middle lobe with a honeycomb network appearance (**Figure 1**). On April 1, a bronchoscopy was conducted, which identified extrinsic compression of the right middle lobe bronchus. Furthermore, endobronchial ultrasound (EBUS) revealed a hypoechoic mass within the lateral segment of the right middle lobe, which was subsequently biopsied. Histopathological examination revealed that the lung parenchyma was replaced by adenoid tumors characterized by adenoid structures lined with columnar epithelium in over 50% of the specimens. Immunohistochemical staining showed CK (+), CK20 (+), CK7 (+), Ki-67 (+, approximately 40%), CDX-2 (−), Napsin A (−), P40 (−), Syn (−), and TTF-1 (−), suggesting a diagnosis of pulmonary enteric-type adenocarcinoma (PEAC) (**Figure 2**).

**Table 1:** Laboratory examination results.

Laboratory	Result	Reference range
White blood cells, ×10^9^/L	5.4	3.5–9.5
Neutrophil, %	76.2↑	40–75
Lymphocyte, %	14 ↓	20–50
Red blood cells, ×10^12^/L	3.8	3.80–5.10
Hemoglobin, g/L	121	115–150
Platelets, ×10^9^/L	247	125–350
Interleukin (IL)-6, pg/mL	5.55↑	0–5.4
IL-10, pg/mL	<1.13	0–12.9
IL-17, pg/mL	3.19	0–21.4
IL-4, pg/mL	<0.85	0–8.56
IL-12, pg/mL	<1.44	0–3.4
Tumor necrosis factor-α, pg/mL	<1.29	0–16
Interferon-γ, pg/mL	<1.65	0–23.1
C-reactive protein, mg/mL	138.00↑	0–8.0
Ferritin, ng/mL	386.00↑	13–150
CEA, ng/mL	1.36	0–6.5
CA199, U/mL	4.96	0–27
CYFRA21-1, ng/mL	3.66↑	<3.3
NSE, ng/mL	10.80	0–16.3

CEA, carcinoembryonic antigen; CA199, carbohydrate antigen 199; NSE, neuron specific enolase.

↑, elevated level; ↓, reduced level.

**Figure 3:**
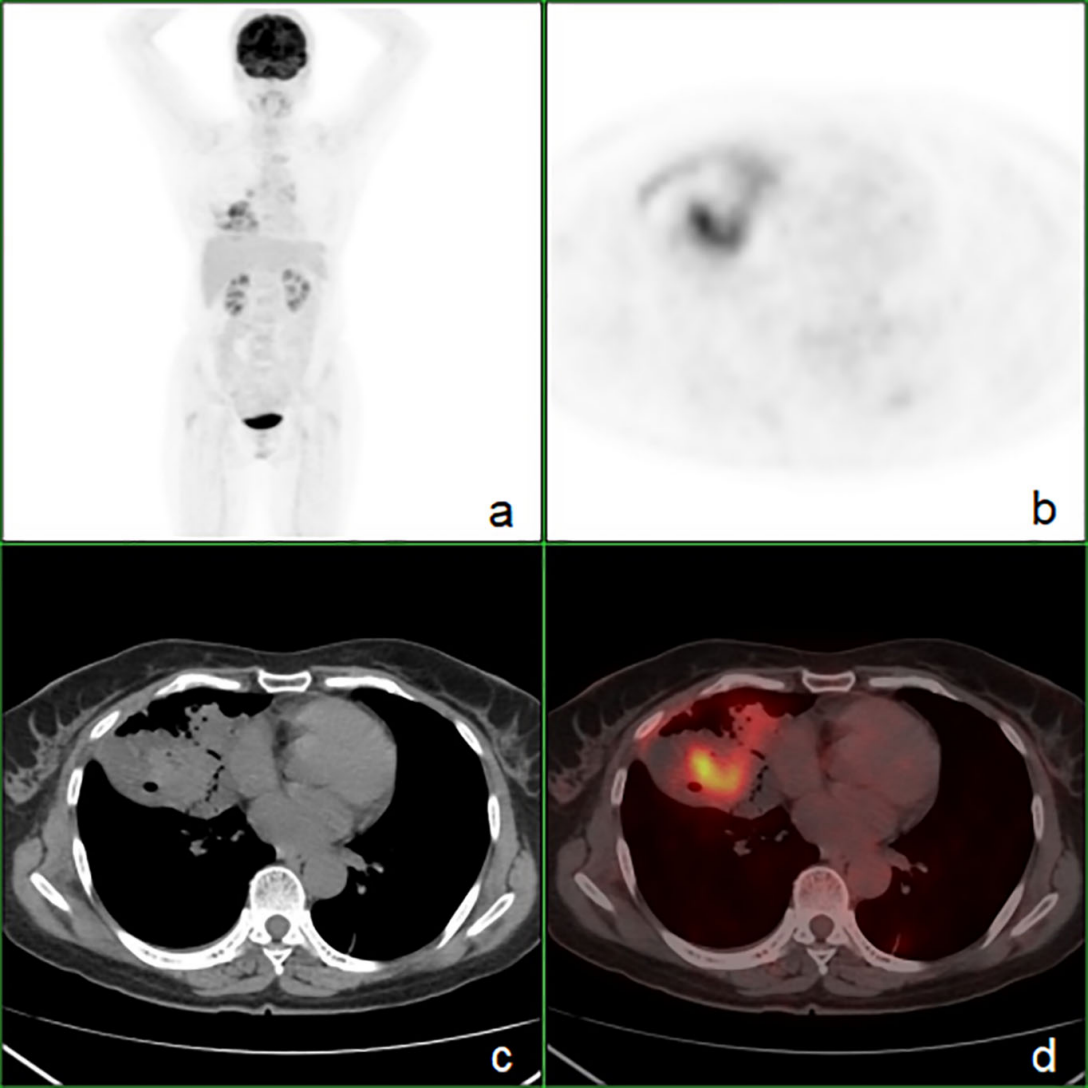
The whole-body ^18^F-FDG PET/CT showing heterogeneous FDG uptake (SUVmax = 8.4) within the right middle lobe and no evidence of hypermetabolic foci in the gastrointestinal tract. **(A)** PET maximum intensity projection map, **(B)** axial PET image, **(C)** axial CT image, **(D)** axial fusion image.

To rule out the potential for lung metastasis from a gastrointestinal primary tumor, the patient underwent a comprehensive evaluation. On April 3, both gastroscopy and colonoscopy were performed, which did not detect any significant pathological findings. Subsequently, on April 4, an ^18^F-FDG PET/CT scan was conducted for further investigation, which identified an ill-defined FDG-avid lesion, measuring approximately 77 mm × 43 mm, with a maximum standardized uptake value (SUVmax) of 8.4, within the consolidative collapse of the right middle lobe. Additionally, an enlarged lymph node was identified in the right hilar region, measuring approximately 11 mm × 17 mm, with an SUVmax of 3.5. No other notable hypermetabolic lesions were observed within the gastrointestinal tract, suggesting that the primary tumor was located in the lung (**Figure 3**).

The patient’s final diagnosis was PEAC (T4N1M0, stage IIIb). Despite the recommendation for anti-tumor therapy, she declined treatment and was discharged on April 6 following the administration of symptomatic care only.

At the 2-month follow-up phone call after discharge, the patient reported a marked worsening of cough and sputum production, along with a 4-kg weight loss, and had not initiated any anti-tumor therapy. The timeline of clinical events from symptom onset to the last follow-up appointment is shown in **Supplementary Figure S1**.

## Discussion

PEAC, first described by Tsao and Fraser in 1991 (7), is a rare primary lung adenocarcinoma with colorectal cancer-like histological and immunohistochemical features (3, 8–10). PEAC is a relatively rare condition, with fewer than 500 cases reported in the English literature, primarily as case reports or small series (1, 3, 4, 8–15). Diagnosis of PEAC necessitates distinguishing it from gastrointestinal adenocarcinoma metastasis (3, 9, 12, 16), as treatment approaches differ significantly. This case report details the clinical course, diagnostic workup, and follow-up of a PEAC patient and aims to increase understanding of the disorder.

PEAC predominantly affects the elderly, with a male predominance (4, 11, 17–19). The relationship between smoking and PEAC incidence remains controversial (11, 19, 20), warranting further study. Clinically, PEAC presents similarly to other primary lung adenocarcinomas, with respiratory symptoms such as cough, sputum, chest tightness, shortness of breath, and the absence of gastrointestinal symptoms (20). Our patient, a 55-year-old female non-smoker, initially presented with inflammatory symptoms. However, the progression of the right lung lesion after anti-inflammatory treatment raised suspicions of an alternative diagnosis.

Imaging of PEAC typically presents as nodules or masses (11, 18, 20), and some cases exhibit imaging findings similar to pneumonia (20). Bian et al. (20) analyzed chest CT data of 13 PEAC patients and 27 lung metastasis patients from colorectal cancer, finding no significant differences in lesion characteristics. The location type of PEAC predominantly exhibits characteristics of a peripheral nature. However, patients with PEAC did not present any ground-glass opacity (11). In our case, PEAC presented as atelectasis with honeycombing changes, a previously unreported finding, demonstrating the diversity of imaging findings.

In our case, bronchoscopy and EBUS play a crucial role in the visualization and biopsy of PEAC lesions, and biopsy via EBUS showed histopathological features of columnar epithelial adenomatous tumors with immunohistochemical characteristics consistent with PEAC. For the diagnosis and differentiation of PEAC, CK7, CDX2, CK20, and TTF-1 are important immunohistochemical markers (11, 14, 17, 18, 21, 22). However, it is crucial to highlight that the differentiation of PEAC from metastatic colorectal cancer should be based on comprehensive clinicopathological correlations, rather than solely on morphological, immunohistochemical, or molecular characteristics (3, 23). Our case’s immunohistochemical staining showed CK(+), CK20 (+), CK7(+), Ki-67(+, approximately 40%), CDX-2 (−), Napsin A (−), and TTF-1 (−).

Exclusion of primary gastrointestinal malignancies is essential, with gastroscopy and colonoscopy serving as vital diagnostic tools (3, 9, 10). PET/CT imaging offers a systemic and highly sensitive approach to tumor detection. It is pivotal in the diagnosis of lung malignancies. Our case, in conjunction with limited prior PEAC cases (15, 24–29) undergoing PET/CT, illustrates that PET/CT is instrumental in identifying malignant tumors, providing accurate staging, and effectively also ruling out primary gastrointestinal adenocarcinoma. This is confirmed through the integration of gastrointestinal endoscopy, including our case. Current data (15, 24, 25, 27, 29) indicate that the SUVmax for PEAC ranges from 2.6 to 12.7, exhibiting considerable variability. To rule out primary colorectal cancer on PET/CT, it is also necessary to differentiate it from physiological bowel FDG uptake. Physiological uptake typically appears diffuse or segmental, whereas the uptake of colorectal cancer is usually focal.

The non-specific clinical and radiological presentations of PEAC necessitate a high index of suspicion to avoid misdiagnosis. In our case, the lack of elevated tumor markers carcinoembryonic antigen (CEA) and CA199, along with symptoms and imaging, initially led to a misattribution to inflammation (11, 16). In fact, pneumonia might coexist with a tumor due to bronchial stenosis causing poor drainage, and the resolution of the left lung shadow on the CT scan after anti-inflammatory treatment also suggests concurrent inflammation and tumor presence.

The final diagnosis of PEAC (T4N1M0, stage IIIb) in this patient was based on tumor size, lymph node involvement, and the absence of distant metastasis. Surgical intervention is preferred for early-stage PEAC, while chemotherapy is the mainstay for advanced stages (30). Despite the generally poor prognosis, some patients achieve remission with radiotherapy and chemotherapy (25). Our patient was recommended anti-tumor therapy, but she declined, just opting for symptomatic care. Follow-up revealed clinical deterioration, highlighting the aggressive nature of PEAC.

In summary, this case report expands the radiological spectrum of PEAC, contributes to the limited literature, underscores the role of ^18^F-FDG PET/CT in diagnosis, and emphasizes the need for a multi-technique approach, providing valuable insights for future clinical management of this disease.

## Data Availability

The original contributions presented in the study are included in the article/**Supplementary Material**. Further inquiries can be directed to the corresponding author.
