# Supplementary material for: Case report: 18F-FDG PET/CT in pulmonary enteric adenocarcinoma
Source: Front Oncol. 2024 Oct 14;14:1447453. doi: 10.3389/fonc.2024.1447453 (PMC11513299; doi:10.3389/fonc.2024.1447453)
Supplement: Supplementary file 1 [file DataSheet1.docx]

|  | **CT:**  Left pneumonia absorption, right lung disease progression,  Obvious improvement of symptoms.  Continue anti-infection | |  | Admission: fever, cough, phlegm, no hemoptysis |  | **Bronchoscopy:**  right middle lobe bronchial external pressure stenosis; **EBUS:**  right mediolateral hypoechoic lesion; **biopsy:**  PECA | |  | **PET/CT:**  hypermetabolic lung lesions, ipsilateral hypermetabolic hilar lymph nodes, no metabolic abnormalities in the gastrointestinal tract | | |  | Follow up:  symptoms worsened with weight loss and did not receive antitumor therapy |
| --- | --- | --- | --- | --- | --- | --- | --- | --- | --- | --- | --- | --- | --- |
|  |  | ↑ |  | ↑ |  | ↑ | |  | ↑ | | |  | ↑ |
|  |  | March 5 |  | March 29 |  | April 1 | |  | April 4 | | |  | June 8 |
| ――――――――――――――――――――――――→ | | | | | | | | | | | | | |
| Late February 2024 |  | March 27 |  | March 31 | |  | April 3 | | |  | April 6 | |  |
| ↓ |  | ↓ |  | ↓ | |  | ↓ | | |  | ↓ | |  |
| Fever,cough,  expectoration.  **CT:**  bilateral pneumonia.  Start anti-infection therapy. |  | Symptoms recurred and worsen, body temperature 38.7℃ |  | CT:  right middle lobe asthenia with honeycomb network | |  | Gastroenteroscopy :  no abnormality | | |  | Refused antitumor  Therapy and was discharged | |  |

**Supplementary Figure 1:** Timeline of clinical events from symptom onset to last follow-up appointment. CT: computed tomography; PEAC: pulmonary enteric adenocarcinoma.
